# 3D nanostructural characterisation of grain boundaries in atom probe data utilising machine learning methods

**DOI:** 10.1371/journal.pone.0225041

**Published:** 2019-11-18

**Authors:** Ye Wei, Zirong Peng, Markus Kühbach, Andrew Breen, Marc Legros, Melvyn Larranaga, Frederic Mompiou, Baptiste Gault

**Affiliations:** 1 Max-Planck-Institut für Eisenforschung GmbH, Max-Planck-Straße 1, Düsseldorf, Germany; 2 CEMES-CNRS, 29 Rue Jeanne-Marvig, Toulouse, France; 3 Department of Materials, Royal School of Mines, Imperial College, London, England, United Kingdom; Pacific Northwest National Laboratory, UNITED STATES

## Abstract

*Boosting* is a family of supervised learning algorithm that convert a set of weak learners into a single strong one. It is popular in the field of object tracking, where its main purpose is to extract the position, motion, and trajectory from various features of interest within a sequence of video frames. A scientific application explored in this study is to combine the *boosting* tracker and the Hough transformation, followed by principal component analysis, to extract the location and trace of grain boundaries within atom probe data. Before the implementation of this method, these information could only be extracted manually, which is time-consuming and error-prone. The effectiveness of this method is demonstrated on an experimental dataset obtained from a pure aluminum bi-crystal and validated on simulated data. The information gained from this method can be combined with crystallographic information directly contained within the data, to fully define the grain boundary character to its 5 degrees of freedom at near-atomic resolution in three dimensions. It also enables local atomic compositional and geometric information, i.e. curvature, to be extracted directly at the interface.

## Introduction

Atom probe tomography (APT) is a microscopy and microanalysis technique that maps the position of millions of atoms from a material in three-dimensions with subnanometer spatial resolution. The technique relies on the effect of intense electric fields to cause the desorption and ionization of the atoms that constitute the surface of a sharp, needle-shaped specimen. The ions are accelerated away from the specimen’s surface by the electric field and collected by a position-sensitive, time-resolved particle detector. The high curvature of the specimen’s tip (radius of curvature in the range of 20–200 nm) makes the electrostatic field highly divergent, and provides a very high magnification, in the range of 10^6^, to the image formed by the impact of the ions on the detector. The elemental identity of each atom is revealed through time-of-flight mass spectrometry [1]. By processing the impact position, the sequence in which ions are detected and each ion’s elemental identity, a three-dimensional point cloud of the original atomic positions can be reconstructed [2]. APT is often used to measure the composition of grain boundaries and associated segregation phenomena [3–6].

When crystalline materials are analyzed, variations in the hit density on the detector often form a pattern that is specific to the crystalline structure and orientation of the specimen [7]. This pattern is due to the faceting of the specimen and associated trajectory aberrations [8, 9]. The appearance of such patterns is usually accompanied by crystallography-related information present within the reconstruted APT data, which can be retrieved and exploited to calibrate a tomographic reconstruction or extract meaningful microstructural information [1].

Microstructural features, such as grain boundaries or precipitates, also lead to variations in the hit density on the detector. These variations are caused by the local magnification effect [2, 10, 11] that appear due to irregularities developing in the local curvature of the specimen. Indeed, as the electrostatic field necessary to provoke the field evaporation depends on the local environment of the evaporating atom [12], the evaporation field of various phases is different, which leads to the appearance of local curvature and hence magnification. Grain boundaries are defective regions in the material’s microstructure, and as such can usually be observed on the detector hit map via the hit density modification they induce locally.

It follows that the information contained within atom probe detector hit maps on 1) the crystallographic orientation of each grain and 2) the position and shape of grain boundaries, can be combined to completely crystallographically define grain boundaries to 5 degrees of freedom [13–15]. This can then be further correlated to local atomic compositional variations [16]. This unique type of analysis can currently only be achieved with APT and it is invaluable to the understanding of nanostructure-property relationships in engineering materials. However, this type of analysis is typically done manually. It is slow and leaves too much room for individual interpretation, making it a source of significant quantification errors and reduced reproducibility.

Here, we explore how machine learning methods can facilitate grain boundary identification and extraction, so as to provide user-independent, automatic and reliable structural characterization, while allowing direct compositional analysis. Tracking moving subjects has been under intensive research for decades. An object tracking algorithm involves locating an object in successive frames of a video or camera. They have been widely applied to video surveillance, autonomous driving or human-computer interaction [17–19]. The problem of object tracking in video can be summarized as the task of finding the position of an object in every frame. Tracking encompasses conceptually similar but technically different ideas, one of which utilizes *boosting* methods, in which the object to be tracked is discriminated by a *boosting* classifier from the background. A *boosting* classifier is a type of machine learning classification technique that creates multiple models and then “boosts” their performance by combining them together [20].

We propose a method based on exploiting the variations in the hit density. Our approach combines the *boosting* tracker with Hough transform algorithm, and principal component analysis (PCA) for automatically extracting the spatial coordinates of grain boundaries. We named this protocol BooT-PCA (Boosting- Tracking-Principal-Component-Analysis). This new approach enables 3D structure and chemical segregation at interfaces to be studied with high precision and efficiency. A pure-Al bi-crystal was chosen for the clarity of its crystallographic features. The application of BooT-PCA algorithm is not only limited to Al-datasets, it can be easily extended to other material systems where density variations in the detector hit map from grain boundaries are readily visible.

## 1 Experimental results

Pellets of commercially pure Al (> 99.99%) were used to form bi-crystals via the Bridgman technique. The crystals were sectioned using a diamond wire saw into rectangular blocks that were 3 mm long, 1 mm wide and 500 *μ*m thick. The Σ3(70.53°) [110](1-1-1) grain boundary was placed end on when looking at the 3x1 mm face, and 45° away from the longest axis of the block. One 3 x 1 mm face was mechanically polished for a few tens of microns using 1200 and then 4000 grit SiC paper. The block was then dipped into an aqueous solution of HNO_3_, HCl and HF to optically reveal the GB. Further detail to the experimental procedures can be found in the literature [21, 22].

APT specimens were fabricated from the grain boundary using an in situ site-specific lift-out method [23] in a FEI Helios dual beam scanning electron microscopy / plasma focused ion beam (SEM / PFIB) instrument with a Xe source. Compared to the conventional Ga FIB, the Xe PFIB show faster material removal rates [24]. More importantly, Ga is known to segregate at interfaces, especially at grain boundaries, during FIB-milling of aluminum and aluminum alloys. Using an Xe PFIB can avoid such ion implantation damage [25, 26]. During specimen sharpening, transmission Kikuchi diffraction (TKD) was employed to locate the grain boundary within the needle-shaped APT specimen [27] using an EDAX electron backscatter diffraction (EBSD) detector and analyzed using the accompanying OIM software (version 7.3.1). APT experiments were conducted using a CAMECA LEAP 5000 XS instrument. It is a straight-flight-path device equipped with a local electrode. Voltage-pulsing mode was used to collect APT data with a pulse fraction of 15% and a pulse repetition rate of 250 kHz. The target detection rate was set at 1 detection event per 100 pulses while the specimen was kept at a base temperature of 60 K during the measurement. The corresponding reconstruction was crystallographically calibrated using the protocol described by [28].

**Fig 1:**
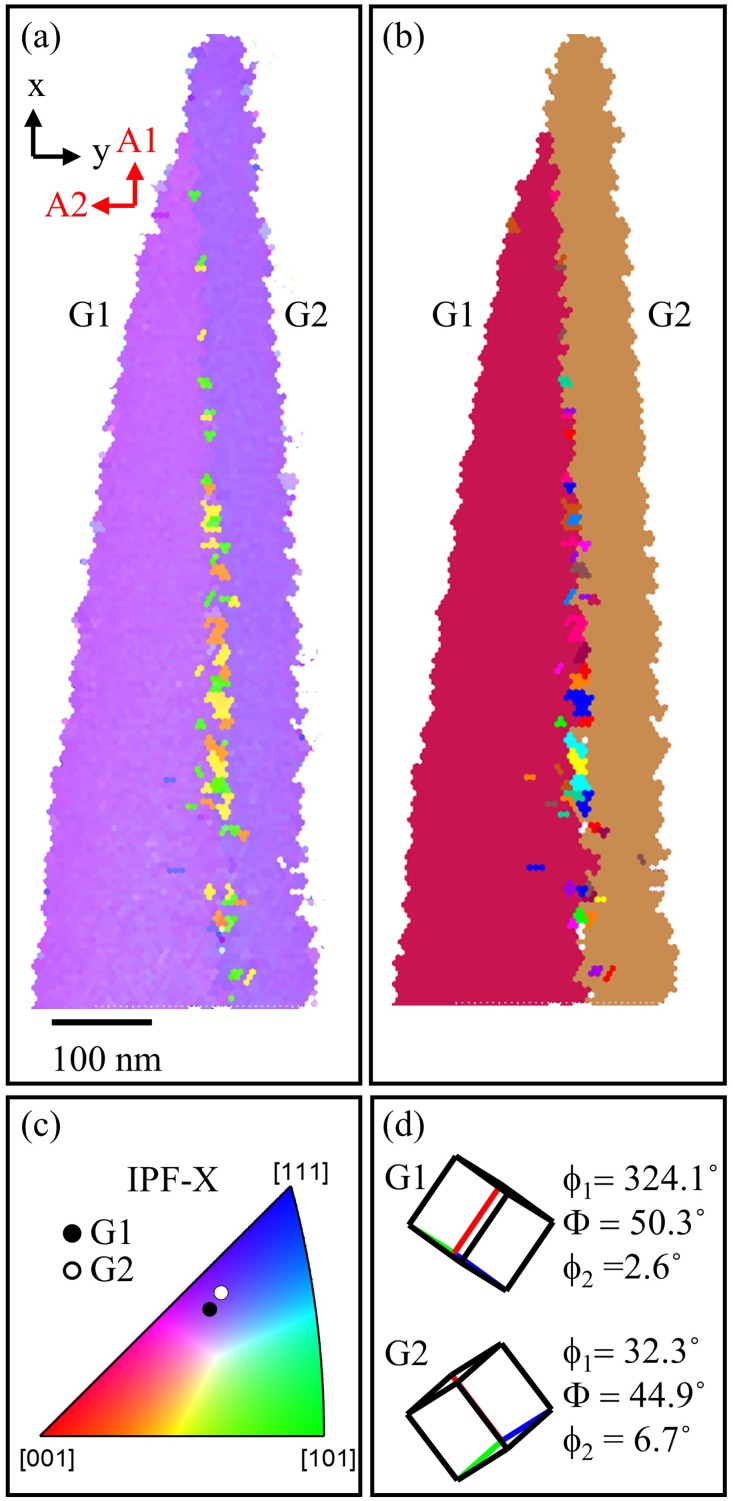
(a) TKD IPF map of the Al bi-crystal atom probe specimen. (b) Unique grain color quick map. (c) IPF-X with crystallographic direction along *x*-axis plotted. (d) Orientation unit cell and Bunge-Euler notation of grains with TKD detector frame.

The TKD results shown in Fig 1 clearly indicate that the grain boundary region was captured in the analyzed specimen and offer an independent experimental confirmation of the crystallographic character of the interface. Fig 1(a) is an inverse pole figure map (IPF) along the arbitrarily defined *x*−direction, which is approximately aligned with the axial direction of the specimen. The A1 and A2 directions show the orientation of the EBSD detector relative to the display view. Grain cleanup was performed using a single iteration of grain dilation in the TSL OIM software with a grain tolerance angle of 5° and a minimum grain size of 2 pixels. A thin noisy region between the two grains can be observed, since the boundary plane is not perfectly orthogonal to the viewing plane. For additional clarity between the two grains, a unique grain color map is provided in Fig 1(b). The color triangle corresponding to the IPF map in Fig 1(a) is shown in Fig 1(c), where the orientation of each grain along this projection is plotted. The unit cell representation of each grain as well as the measured Bunge-Euler notation are provided in Fig 1(d). The disorientation in angle/axis pair representation [29] was measured to be 59.3°/[0.590, 0.575, 0.568].

**Fig 2:**
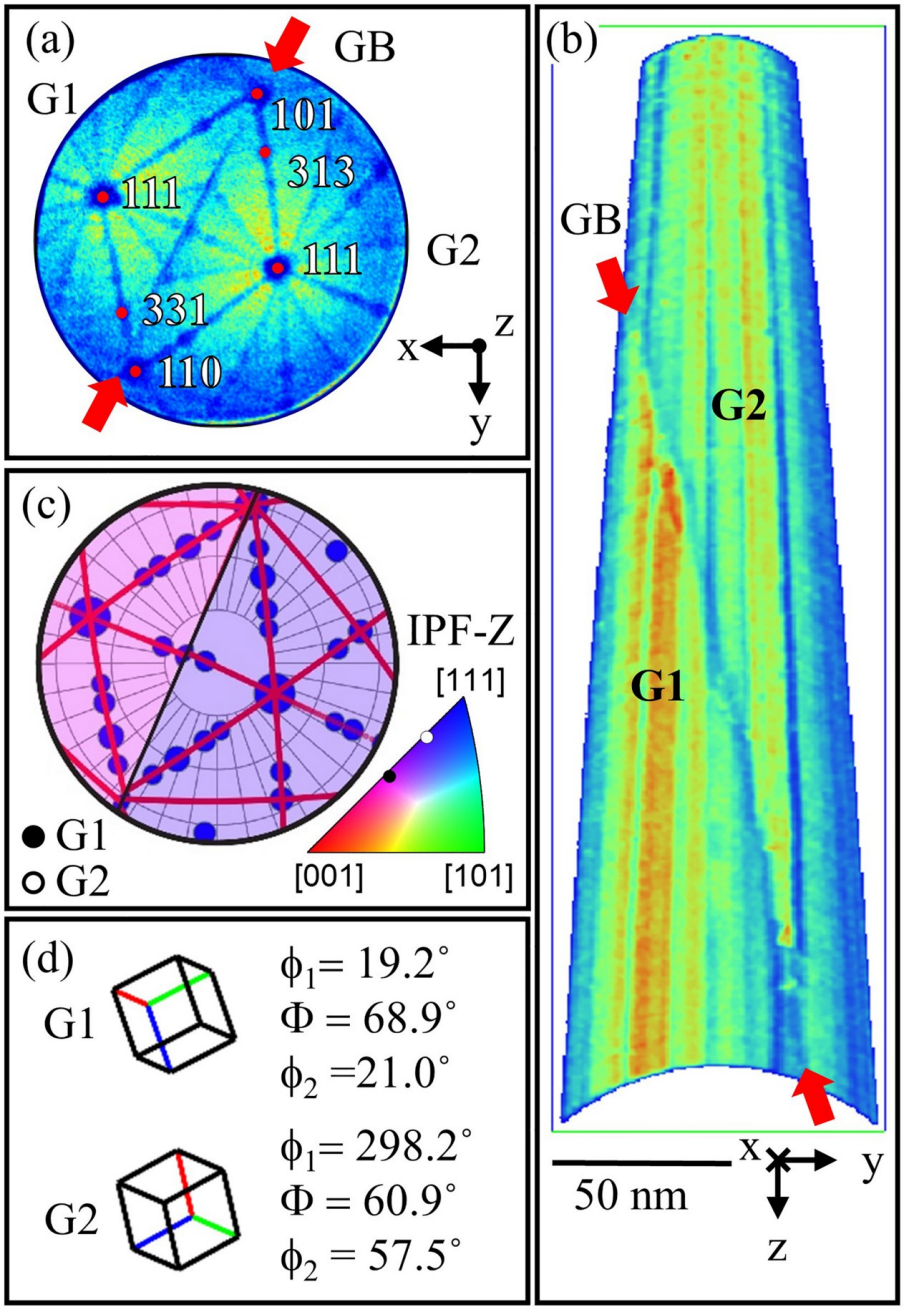
**(a)** FDM of experimental APT data of Al bi-crystal with crystallographic patterns indexed. **(b)** Al-density map of reconstruction, ≈ 1.2 nm thick, through the centre of the reconstruction on the YZ-plane. **(c)** Stereographic projection representation of the two grains, showing low index poles and zone-lines, colored corresponding to the IPF-Z. **(d)** The orientation cells and Bunge-Euler notation within the APT detector frame.

The same specimen was subsequently analyzed using APT. A detector hit map of 1 million sequentially collected ions from the centre of the dataset is shown in Fig 2(a). The low density features in the map clearly capture crystallographic information from each grain as well as the GB. The dominant crystallographic poles were compared to that in a stereographic projection of the crystal and have been indexed accordingly. 48.3 million ions were collected in total and reconstructed using IVAS. Crystallographic calibration [30] was performed so that the resulting reconstruction was as accurate as enabled by the protocol implemented in the commercial software. The image compression factor (ICF) and field reduction factor (*k*_*f*_) were found to be 1.53 and 4.92 respectively. In order to more clearly show the boundary in the reconstruction, a density map, shown in Fig 2(b), was calculated for a 0.5nm wide slice cutting through the entire reconstruction along the *x* = 0 plane. The inherent crystallographic information contained in Fig 2(a) was then used to directly calculate the orientation of each grain relative to the APT detector. Fig 2(c) is a stereographic projection representation of each grain. Each grain is colored according to the orientation within the IPF-Z color triangle. The colors are close to those used for the TKD IPF map but are not exactly the same due to the slightly different physical orientation of the needle within the atom probe compared to the EBSD setup. Fig 2(d) are the corresponding unit cell and Bunge-Euler notation of each grain. The disorientation angle/axis pair, which is independent of the specimen frame, was then calculated from the APT measurements and found to be 59.4°/[0.624, 0.555, 0.550] and is in excellent agreement with that measured from TKD.

The normal vector to the boundary was then measured manually within the atom probe reconstruction. Nine arbitrary points on the GB were recorded (by selecting points at the centre of the low density region of the GB at different z values) and the average normal vector (in the specimen frame) between these points (*n*_*h*_) was computed:

$$n_{h}=\left[-0.87,0.44,-0.12\right]$$ (1)

## 2 Field evaporation simulations

**Fig 3:**
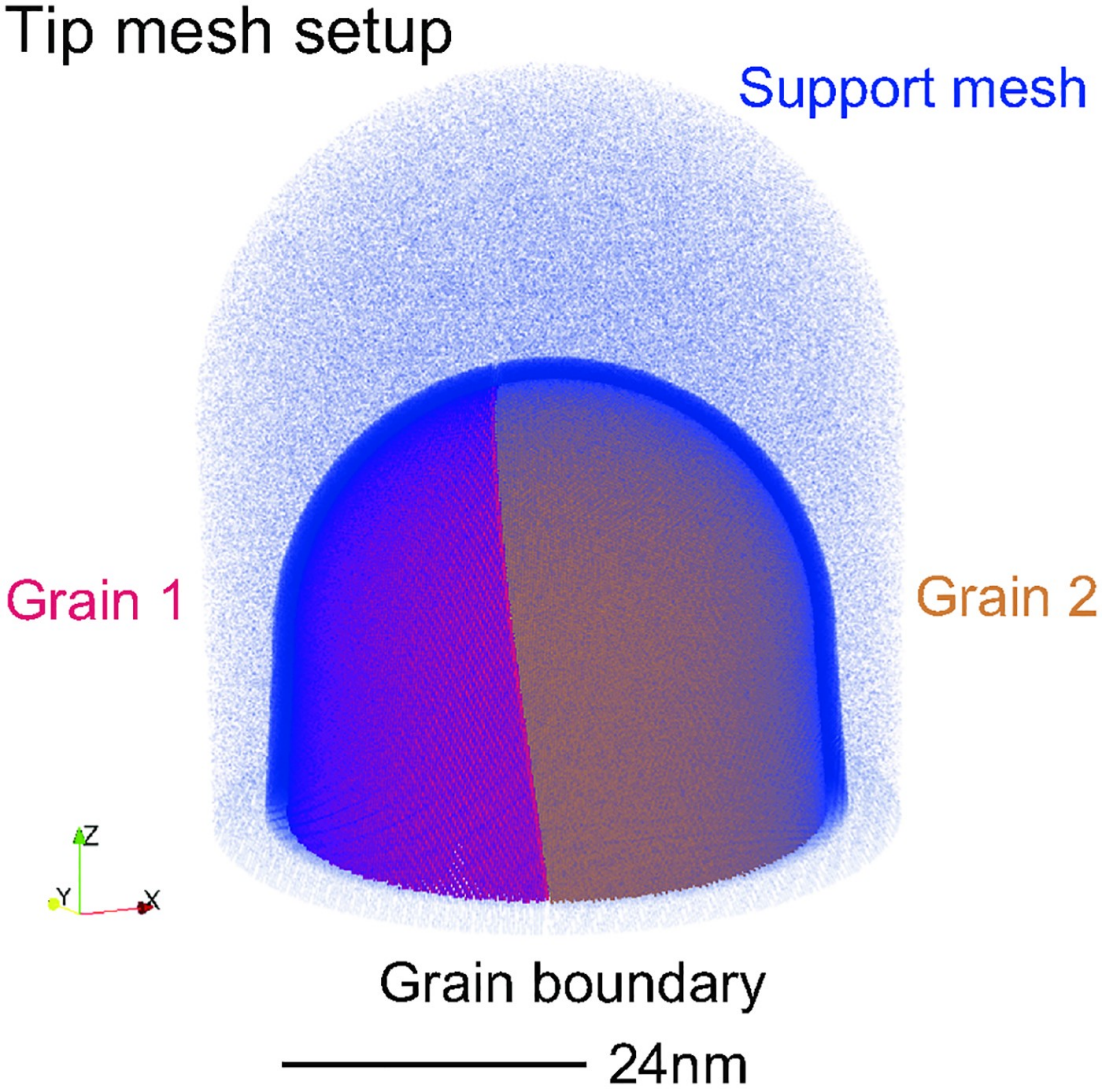
The experimentally informed simulation setup for direct comparison with experiment.

To verify the applicability of BooT-PCA, we initially ran a test with a simulated bi-crystal, the geometry of which was based on that observed in the experimental data. Specifically, the orientation of each grain relative to the detector, as well as the position and normal of the grain, matched that observed in the experimental results (Fig 3).

TAPSim [31] was then used to simulate the field evaporation process. As detailed in [32–34], this requires the definition of a cascading mesh of integration points. At its finest scale, the mesh resolves the nanoscale dimensions and atomic structure of the specimen. At progressively coarser scales, it bridges to the centimeter-sized chamber of the atom probe. Fig 3 displays only the three finest scales of this composite mesh: the specimen itself, a finer mesh corresponding to a transition zone with a thickness of 2 nm to accurately capture the ion’s launch trajectories, and an order of magnitude more coarsely sampled support mesh.

The virtual specimen was synthesized as a 15 nm high conical frustum with 23.6 nm base radius and 4.5° shank angle onto which a 15 nm sized hemisphere was placed. Atomic positions therein were defined by inscribing two rotated aggregates of aluminum (face-centered cubic, 0.404 nm) crystal unit cell lattices. Their orientation was assigned based on the values measured on the bi-crystal by TKD performed directly on the APT specimen (see Fig 1). In Bunge-Euler notation their orientation are *φ*_1_ = 324.1°, Φ = 50.3°, *φ*_2_ = 2.6° for grain 1 and *φ*_1_ = 32.3°, Φ = 44.9°, *φ*_2_ = 6.7° for grain 2. The virtual bi-crystal synthesis was completed by cutting the two crystals along a 0.2 nm-thick plane with a unit normal [−0.87, 0.44, −0.12] corresponding to the experimentally measured value. Given that the purpose of this setup was to test the application of the boundary tracking algorithm, rather than infer the detailed atomic structure of the grain boundary, the structure generated here was not atomically relaxed. The resulting mesh contained a total of 2.9 × 10^6^ Al atoms. These were encapsulated in a support mesh comprising 3.5 × 10^6^ integration points. Field evaporation was simulated assuming (+1) as the evaporation charge state and 19 × 10^9^ V m^−1^ field strength, based on image hump model considerations [35]. Appendix 6 provides further technical details.

## 3 Introducing BooT-PCA

### 3.1 Context

In APT, we have two distinct coordinate systems, namely, the specimen space and detector space. The former is defined as the three-dimensional Cartesian coordinates of the reconstructed atoms, whereas the latter is defined as the ion impact position on the detector. Detector hit maps are 2D histograms of the raw ion impact positions on the detector, where the color indicates the number of ions which have hit the detector within a two-dimensional bin, i.e. pixel. The detector hit coordinates are contained within a data file (usually .*epos*), which also contains their corresponding 3D coordinates within the tomographic reconstruction. Due to their intrinsically different field evaporation behaviour that causes slight trajectory aberrations, microstructural features appear as variations in the hit density. Once a feature has been located on the detector, it should be possible to filter out the points that are associated to this specific feature.

**Fig 4:**
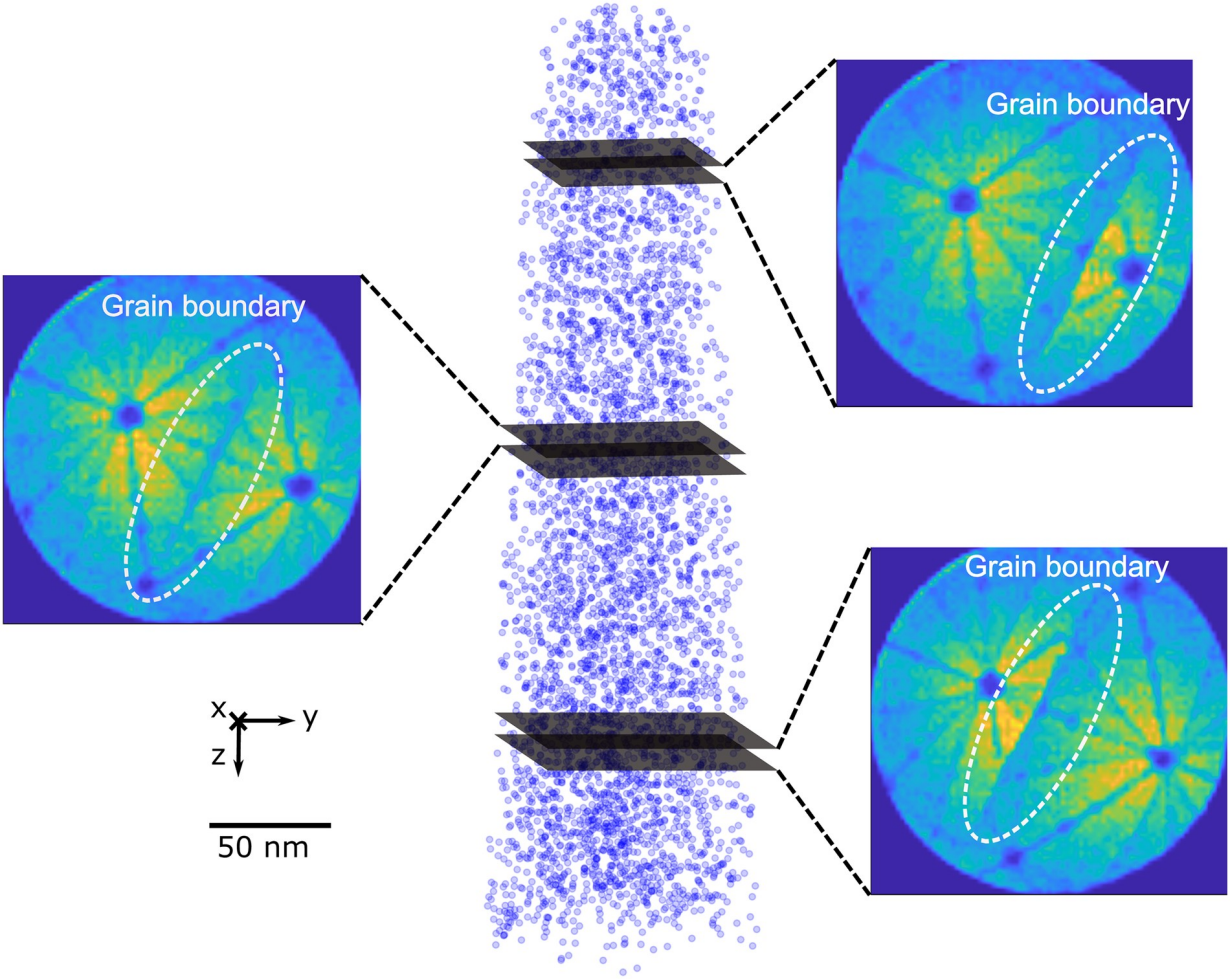
Grain boundary is observed in a series of detector hit map images. The goal is to follow the movement of grain boundary and reconstruct it in 3-D Cartesian space.

In cases where the specimen contains one or more features, one can usually observe their trace as moving objects in a sequence of detector hit maps calculated for a finite number of ions, e.g. typically .5 to 1 × 10^6^ ions. This is demonstrated in Fig 4) where the position of the GB line migrates across the detector hit map at different stages of the experiment, corresponding to different depths in the resultant reconstruction. To reconstruct a specific line feature automatically in 3D, an iterative procedure which reads through successive sections of the dataset is required.

The problem of object tracking in video can be summarized as the task of finding the position of an object in every frame. Tracking encompasses conceptually similar but technically different ideas, one of which utilizes *boosting* methods, in which the object to be tracked is discriminated by a *boosting* classifier from the background. A *boosting* classifier is a type of machine learning classification technique that creates multiple models and then boosts their performance by combining them together [20].

**Fig 5:**
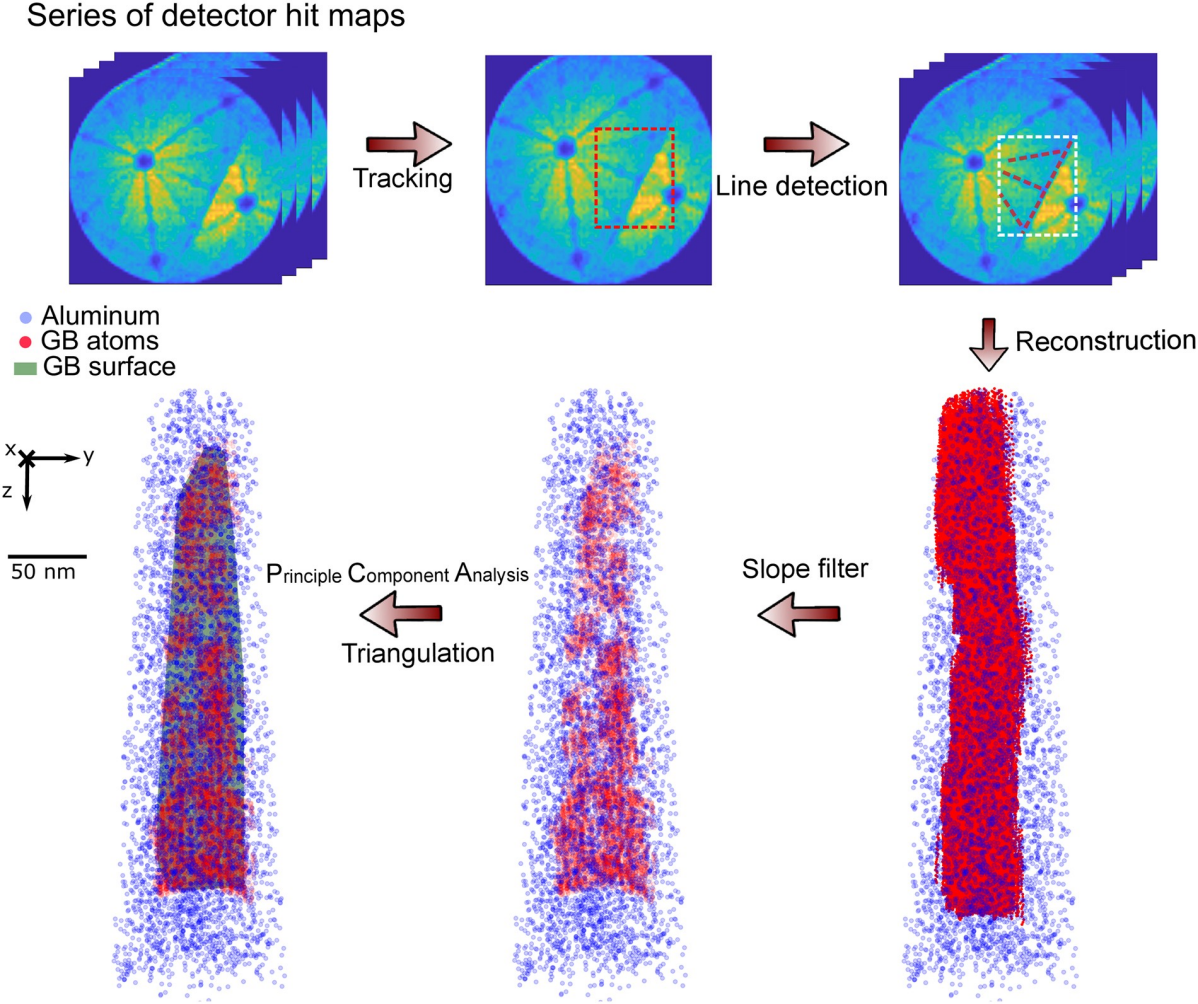
A flowchart of the BooT-PCA on an aluminum bi-crystal dataset: Firstly, image processing is performed on a series of detector hit maps, secondly tracking and line detection are performed. Next the collected data is reconstructed into 3D Cartesian coordinates, thirdly, a slope filter, PCA and triangulation (consists of mesh vertices) are applied sequentially to filter out zone lines atoms and fully reconstruct the GB surface.

The BooT-PCA method is based on the possibility to track a grain boundary observed as density variations in successive detector hit maps (see Fig 4), in order to extract the position and mass-to-charge ratio of the atoms in the specimen’s space that belong to this specific grain boundary. Because the commercially available software allows to routinely retrieve RGB (red-green-blue) detector hit maps, the input in BooT-PCA is a series of such images instead of the raw detector hits. BooT-PCA combines the *boosting* tracker and principal component analysis (PCA) for automatically extracting the spatial coordinates of grain boundaries. We show here how additional filters can be introduced if necessary, with an example of a filter based on the Hough transform. The general framework of BooT-PCA is laid out in the flowchart displayed in Fig 5.

### 3.2 Pre-processing

Before introducing machine learning techniques, we developed a feature selection method to ensure that the computer could efficiently learn to track grain boundaries. In the present dataset, the main crystallographic features in detector hit maps comprise poles, zone lines and grain boundaries traces. In order to track the grain boundaries efficiently, we start with reducing the noise (random fluctuation of pixel value in the detector hit map), which can be achieved by convolution of filters with the image [36].

In BooT-PCA, we use two filters to reduce the noise level in the detector hit map, first, a median filter (size: 7×7) followed by a Gaussian filter (size: 7×7). The median filter computes the median of all the pixels inside a specified area and replaces the central element with the median value. A Gaussian filter assigns a weighted average to each pixel from a Gaussian distribution of surrounding pixel intensities. The filter size was selected after a careful manual optimization process. The noise reduction procedure may need to be adjusted, based on measurement conditions, more information on the adjustment criteria can be found in Ref. [37].

### 3.3 *Boosting* tracker

The *Boosting* classifier is very popular in computer vision, showing solid performance in detection and recognition tasks. The first *boosting* algorithm was proposed over two decades ago [20]. It refers to the combination of multiple ‘weak classifiers’ into a single ‘strong classifier’. A ‘weak classifier’ is a classifier that performs slightly better than random guessing. Random guessing is equivalent to a 50% accuracy on a binary classification task.

**Fig 6:**
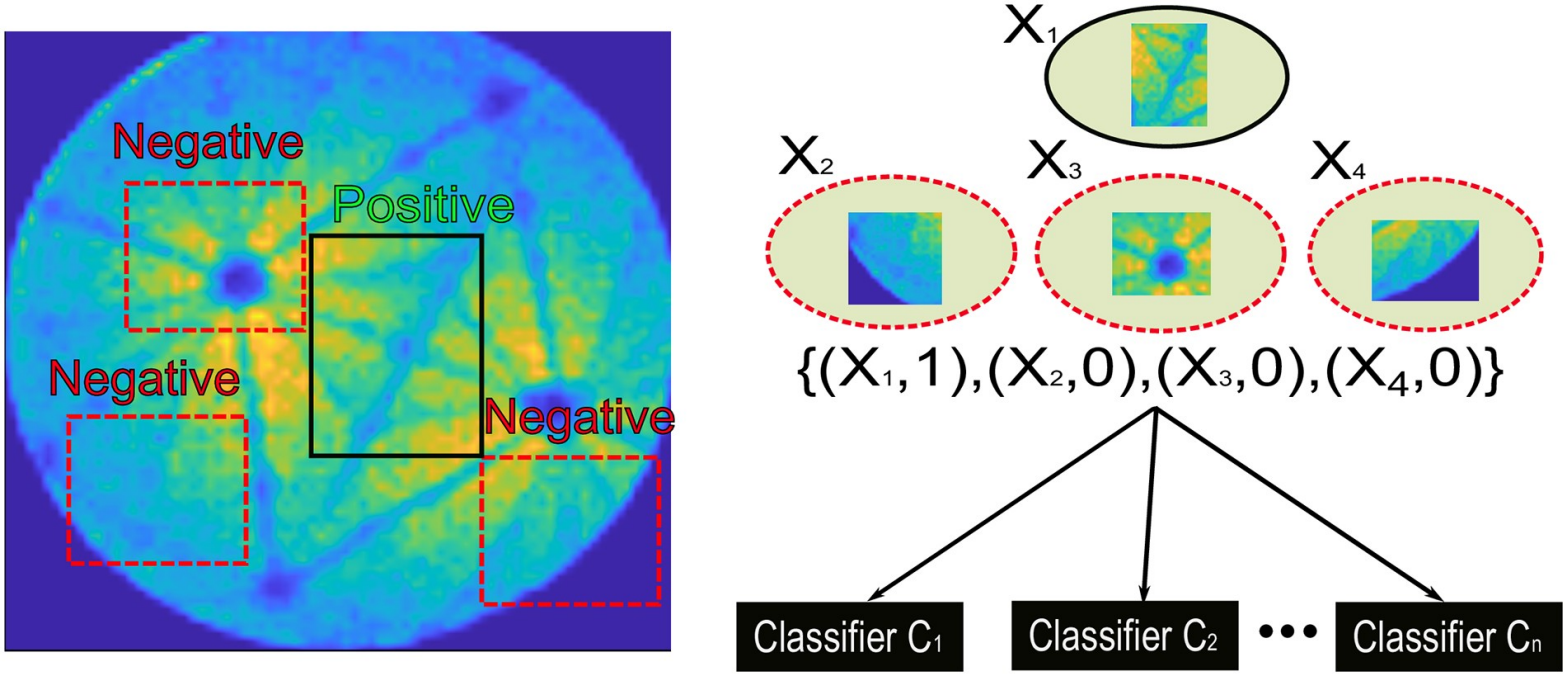
Principle of the *boosting* tracker: At each frame, positive and negative samples are collected and fed into the *boosting* algorithm to create a strong classifier, then the classifier predicts the position of the target in the next frame. (**X**_*n*_, **1**/**0**) indicates group n that contains either positive (1) or negative (0) samples.

The standard protocol [20] of the *boosting* method is illustrated in Fig 6 and the Pseudocode is outlined below in Algorithm. 1. The algorithm takes as input a training set (*x*_1_,*y*_1_), (*x*_2_, *y*_2_),…, (*x*_*m*_, *y*_*m*_) where each (*x*_*i*_) belongs to a domain space *X*, and each label *y*_*i*_ is in label set *Y*. Here we assume Y = {−1, +1}. A key idea of the algorithm is to maintain a distribution of weights over all examples in the training set. The weight of this distribution on training example *i* on round *t* is denoted as *D*_*t*_(*i*).

Initially, all weights are set equally as 1/*m*. During the training period, the weights of incorrectly classified examples are constantly updated at each round, such that the weak learner is forced to focus on the hard examples in the training set. The weak learner’s job is to find an appropriate weak classifier *h*_*t*_: *X* → {−1, +1} for the distribution *D*_*t*_. The goodness of a weak classifier is measured by its error:

$$\epsilon =Pr_{i∼D_{t}}\left[h_{t}\left(x_{t}\right)\neq y_{i}\right]=\sum_{i:h_{t}\left(x_{i}\right)\neq y_{i}}D_{t}\left(i\right)$$ (2)

**Algorithm 1** Adaptive *boosting* method

1. Given: (*x*_1_, *y*_1_),…,(*x*_*m*_, *y*_*m*_) where x_*i*_ is the *i*th sample, y_*i*_ ∈ {−1, +1}, *m* is the total number of samples.

2. Initialize: *D*_1_ = 1/*m*.

3. For t = 1,…,T (t is the number of classifier and T is the total number of classifiers):

 • Train weak learner using distribution D_*t*_.

 • Get weak classifier *h*_*t*_: Ω → {−1, + 1}.

 • Aim: select *h*_*t*_ with low weighed error:

$$\epsilon =Pr_{i∼D_{t}}\left[h_{t}(x_{t})\neq y_{i}\right]$$

 • Update, for *i* = 1, …, *m*:

$$D_{t+1}(i)=\frac{D_{t}(i)exp(-\alpha_{t}y_{i}h_{t}(x_{i}))}{Z_{t}}$$

Where *Z*_*t*_ is a normalization factor (chosen so that D_*t*+1_ will be a distribution) and *α*_*t*_ is weight for the learner, which is mathematically defined as $\frac{1}{2}*\text{ln}(\frac{1-\epsilon }{\epsilon })$.

4. The final strong classifier is defined as:

$$H(x)=\text{sign}(\sum_{t=1}^{T}\alpha_{t}h_{t}(x))$$

When the weak classifier *h*_*t*_ and its corresponding weight are received, *boosting* choose a parameter *α*_*t*_ (as shown in the pseudocode) to measure the importance that is assigned to *h*_*t*_. It is noteworthy that *α*_*t*_ gets larger as *ϵ*_*t*_ gets smaller. The distribution *D*_*t*_ is then updated using the rule shown in the pseudocode. The aim of this procedure is to increase the weight of misclassified examples (by *h*_*t*_), and to decrease the weight of correctly classified examples. In such a way, the hard examples are gaining more weight from the learner. The final hypothesis H is calculated as a weighted majority of the T weak classifiers where *α*_*t*_ is the weight assigned to *h*_*t*_.

In the following, we detail each step in our present work:

1. The training set contains *m* samples where all *x* inputs are patches from the images and y outputs are an element of a set comprising of only two values, 1 (The grain boundary) and -1 (others).
2. Initialize all weights of the samples to 1 divided by the number of patches (*m*).
3. From *t* = 1 to *T* classifier, fit it to the training data (where each prediction is either -1 or 1) and select the classifier with the lowest weighted classification error.

In the context of object tracking, the classifier is trained during runtime (shown in Fig 6). The trained classifier predicts the position of the target in the next frame till the end of video. The relevant package is already available in the open-cv python package [38].

### 3.4 Feature detection and selection

**Fig 7:**
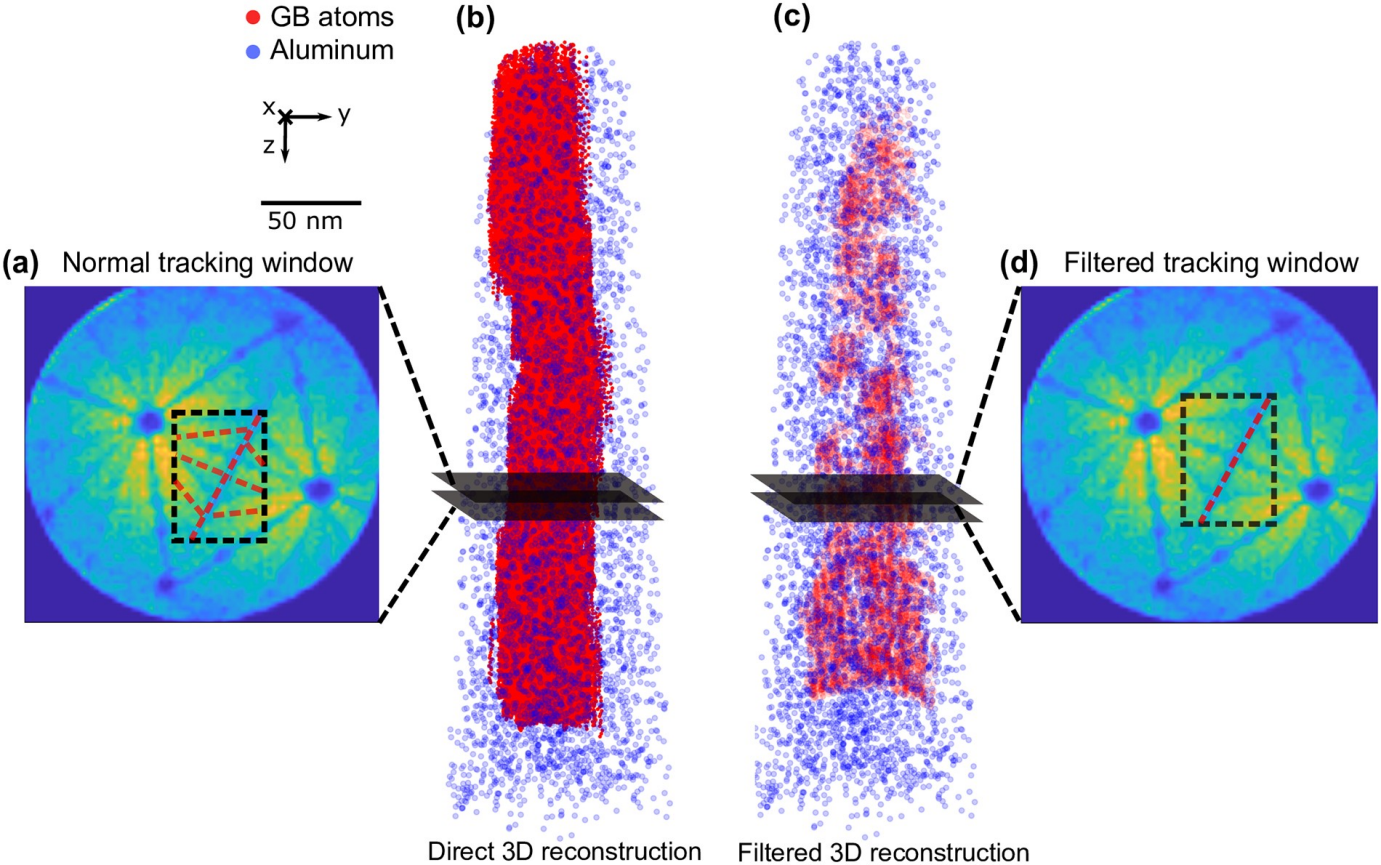
**(a)** A tracking window is user-defined (dotted black rectangle); Hough transformation detects all the features within the frame; Dotted white lines indicate the detected grain boundary and black dotted lines indicate the zone lines. **(b)** Direct 3D reconstruction is performed based on the coordinates in **(a)**; Blue points represent the atoms whereas red represent the extracted ‘GB’ points. **(c)** Refined 3D reconstruction with the slope filter. **(d)** After the slope filter is implemented, the lines with slope smaller than 2 are removed.

The *Boosting* tracker requires the user to define a subset of the image on the first frame that will then be tracked. An example is shown in Fig 7(a) as a red dashed rectangle. This region should contain the region of interest (ROI). Our ROI here is the grain boundary, which appears as a low density linear region on the detector hit maps. Sometimes a part of the grain boundary is not tracked due to the limited size of tracking window. Whilst the size of the tracking window is fixed whilst the size of image of the GB on the detector can change. Therefore if the image of the GB grows too large, the tracking window might not be able to cover it. Even if some parts of the GB have not been identified by *Boosting*, it can be partly solved by the fitting of the GB plane in the following steps of the algorithm. In addition, data extracted from tracking this complete window would not just contain the GB but also the surrounding matrix. Hence, additional filtering steps in BooT-PCA are required so as to only select the feature of interest within the ROI.

Here we aim to track a nearly linear feature, which are known to be efficiently detected by the Hough transform. The Hough transform is widely used in image analysis and computer vision [39]. It is commonly used for indexing Kikuchi patterns collected using electron back-scattered diffraction (EBSD) [40]. It has also been used previously in three-dimensions to reveal the presence of atomic planes within the atom probe reconstruction [14, 41]. In this work, the Hough transform implementation is based on Python open-cv package [38]. Fig 7(a) shows that linear features can be detected using the Hough transform algorithm within the tracking window, and hence used to refine the feature selection inside the ROI.

As shown in Fig 7(b) we collect all points along the detected linear features (red-dotted lines) using Hough transform, then direct 3D reconstruction is performed. However, here, not every linear feature detected in the ROI is necessarily a section of the grain boundary trace. The strong patterning associated to the field evaporation of pure-Al results in several zone lines being present beside the grain boundary line in the tracking window. As a result, the direct reconstruction appears to be in disorder. On the other hand, zone lines have a different slope in comparison to the GB line. We can therefore improve the reconstruction process by implementing a slope-based filter, which discards the lines whose slopes are significantly different from that of the GB line (shown in Fig 7(d)). The filtered data is displayed in Fig 7(c), and reveals a planar feature that corresponds to the GB itself.

### 3.5 Principal component analysis

**Fig 8:**
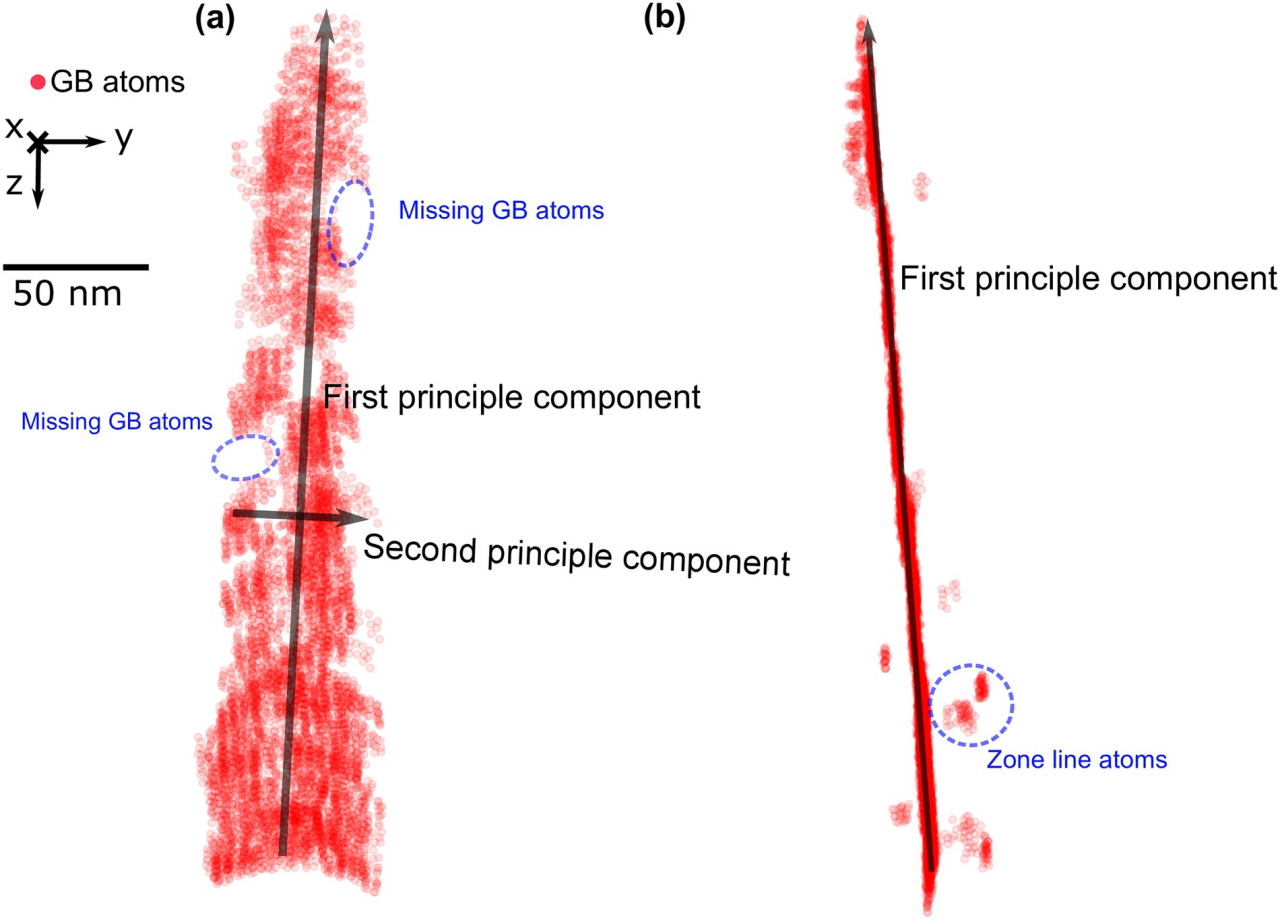
Visualization of GB atoms and principal components. **(a)** Front view of GB atoms, first and second principal vector are plotted, on this two components the variance are the highest. Additionally some GB atoms are missing mainly due to the limited size of tracking window. **(b)** Side view of GB atoms, a few zone line atoms can be seen as well.

We introduce principal component analysis (PCA) to extrapolate the complete grain boundary surface. PCA is a statistical method which simplifies the complexity in high-dimensional data while retaining trends and patterns [42]. Applying PCA on our 3D point cloud, formed by all detected GB atoms, gives us a 3*3 matrix consisting of three eigenvectors and their corresponding eigenvalues. These three eigenvectors have also been referred to as three principal components, which are perpendicular to each other. Among them, the variance of the positions of all GB atoms along the first principal component is the largest, and along the third component is the smallest. In the case of the aluminum bi-crystal dataset, the final GB plane is set perpendicular to the third principal component and positioned at the average position of all the GB atoms. Fig 8(a) and 8(b) are two orthogonal views of the points extracted from the experimental dataset following the application of BooT-PCA.

### 3.6 Grain boundary reconstruction

The set of GB atoms illustrated in Fig 8(a) is not able to fully represent the grain boundary, even if they are mainly located at or in the vicinity of the grain boundary. Therefore, a final step is introduced in the protocol to estimate a realistic GB surface. We set up a fine triangular mesh on the GB plane obtained before. The distance between the mesh vertices is set to 3 nm so that all GB atoms are included. The coordinates of each triangle patch are adjusted to the center-of-mass position of the GB atoms within 3 nm, similar to what was proposed previously in the literature [6, 43]. This final step allows us to finalize the identification of the grain boundary and perform further analyses, such as the measuring its local normal, local curvature, and, importantly, local composition.

## 4 Results

### 4.1 Method verification

**Fig 9:**
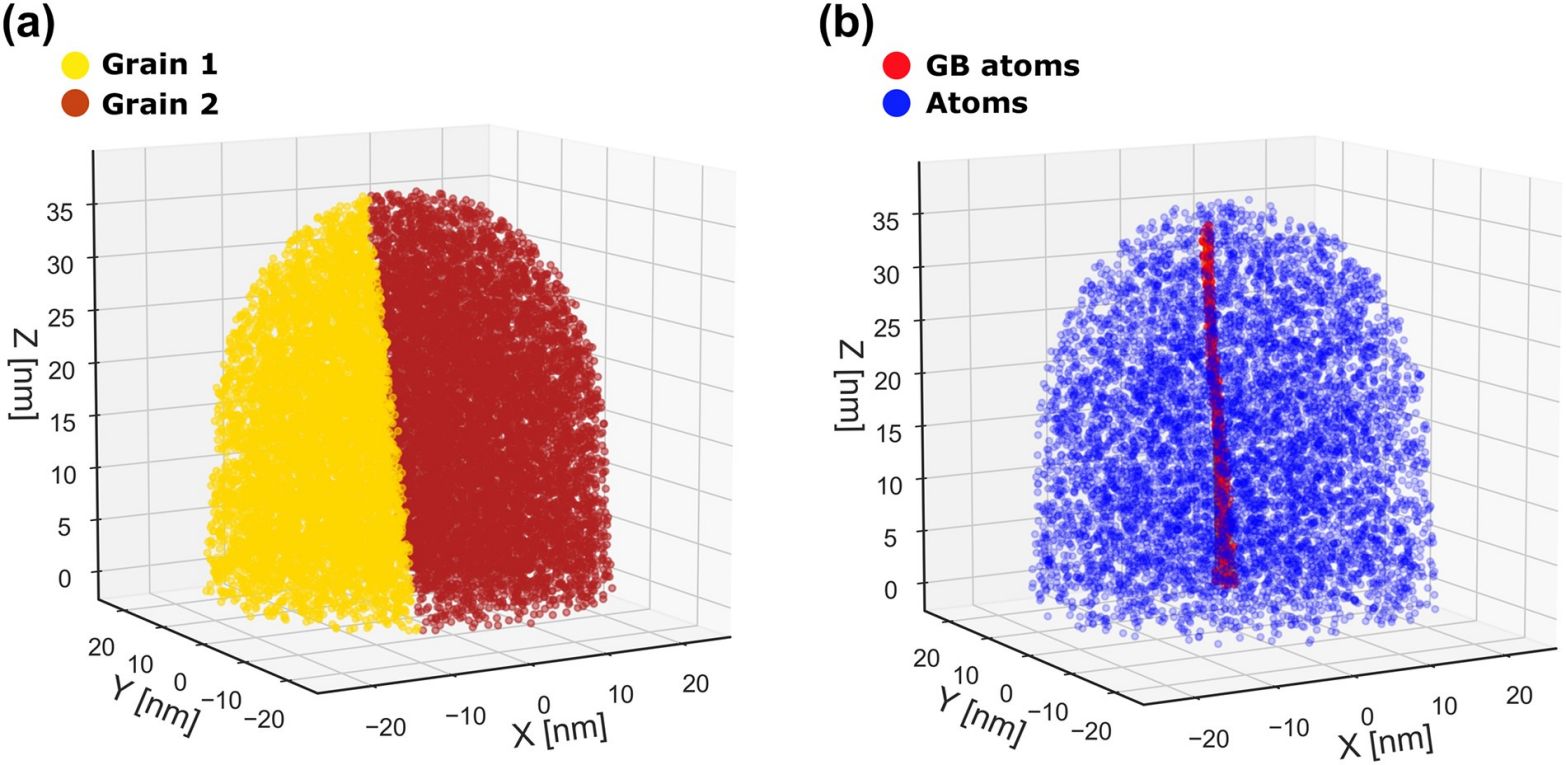
**(a)** TAPSim specimen geometry. The grain boundary normal is set to *n*_*h*_ (Eq 1). **(b)** Reconstruction from TAPsim detector hit map using BooT-PCA.

Fig 9(a) shows the TAPSim specimen input geometry. The normal vector of the GB resulting from the application of BooT-PCA to the simulated detector hit maps is:

$$n_{t}=\left[-0.86,0.47,-0.20\right]$$ (3)

**Fig 10:**
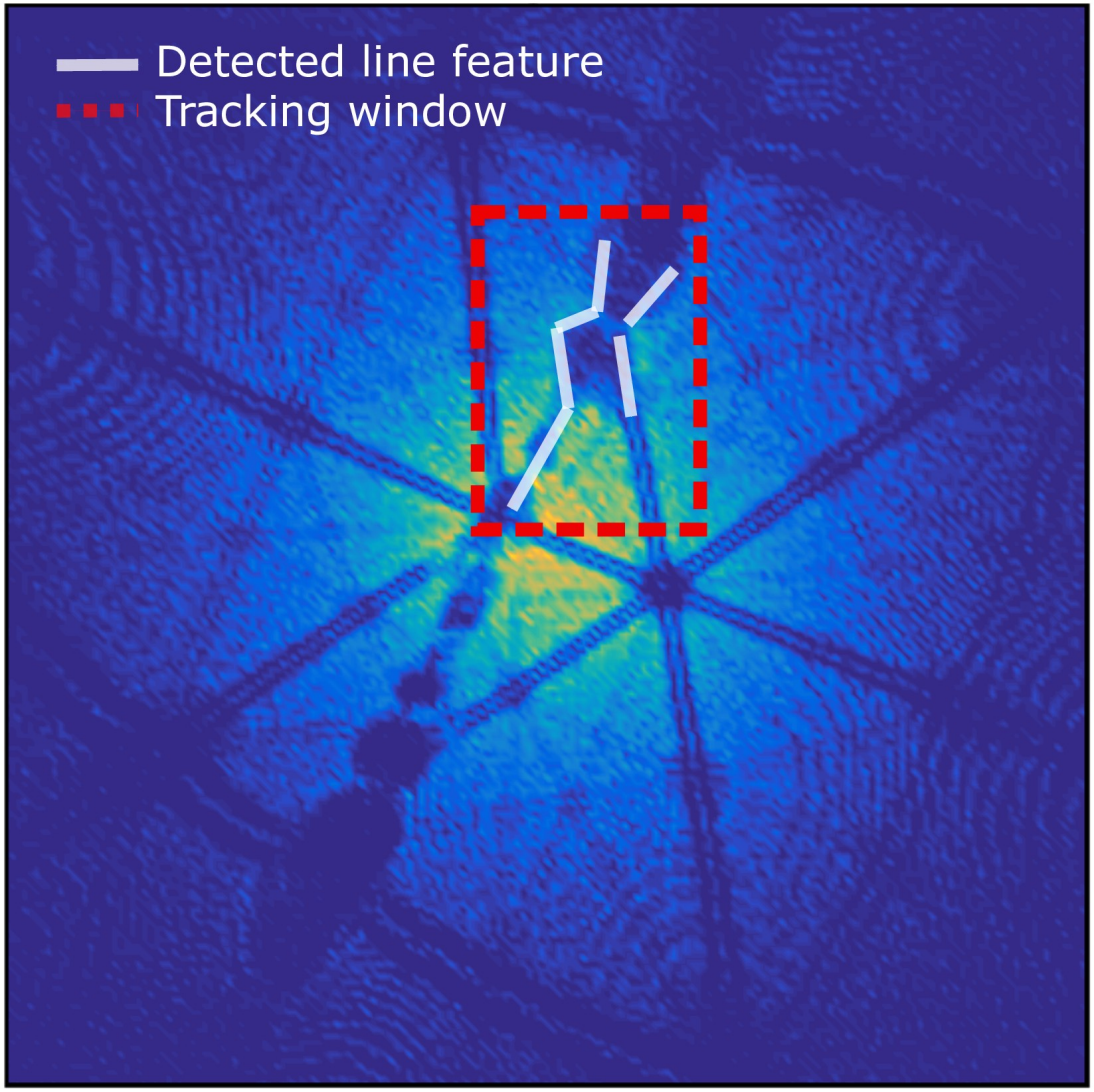
TAPSim-based detector hit map: The lines detected (shown in white) are quasi-linear local ion density fluctuations which the Hough transform picks up. These are affected by the geometry, e.g. the thickness of the boundary, the atomic structure of the adjoining crystals, and known TAPSim field evaporation artifacts [11].

The reconstructed boundary atoms are shown in Fig 9(b). The angular deviation from *n*_*h*_ (Eq 1) is calculated to be 4.6°, which seems acceptable considering the many possible sources of error, including from the simulations themselves as indicated in Fig 10.

### 4.2 Application to the experimental dataset

**Fig 11:**
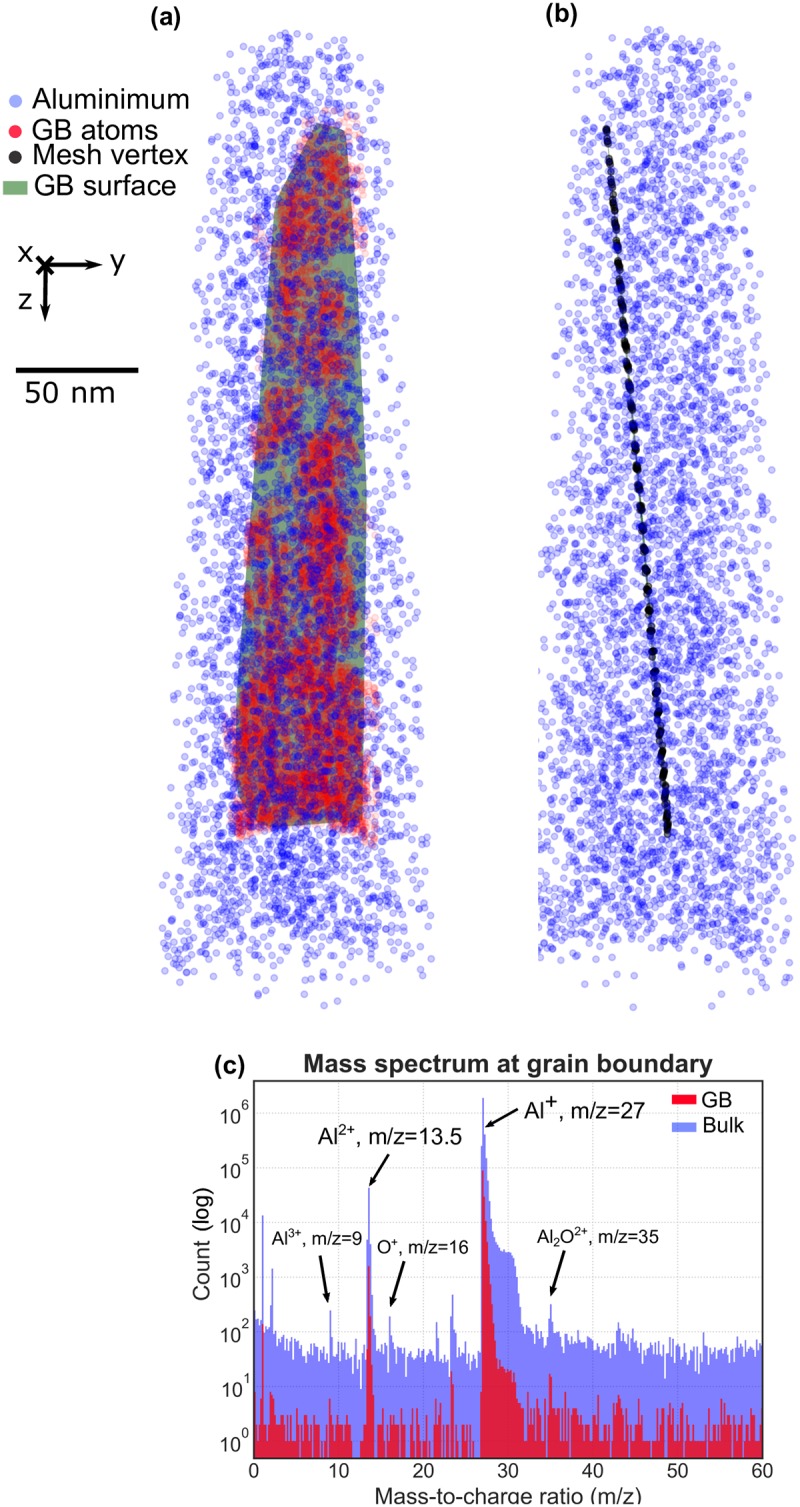
Visualization of GB atoms and surface after the PCA; Red and blue dots indicate the GB atoms and non-GB atoms respectively. **(a)** Front view of GB surface. **(b)** Side view of GB surface. It can be observed that the surface is not perfectly flat. **(c)** Mass spectrum of the GB, the majority of ions are Al^2+^ or Al^+^; In contrast to the spectrum of the bulk, the peak of O^+^ is absent.

The final result is shown in Fig 11(a). The side view of this realistic GB surface is provided in Fig 11(b). A plane equation is fitted to the grain boundary and its surface normal *n*_*m*_ is:

$$n_{m}=\left[-0.87,0.49,-0.11\right]$$ (4)

The discrepancy between *n*_*h*_ and *n*_*m*_ is only 2.9°.

Furthermore, around 140000 GB atoms were selected based on the coordinates of the mesh vertex, from which a mass spectrum (mass-to-charge-state-ratio spectrum, often referred to simply as ‘mass spectrum’) was calculated, which is plotted in Fig 11(c), alongside a mass spectrum for the entire dataset. Analysis of the mass spectrum extracted from the grain boundary provides direct information on its specific chemical composition. The signal-to-background ratio in the GB mass spectrum is also relatively higher compared to bulk (for example at peak 13.5 Da, Signal-to-background ratio of the GB mass spectrum is 5:1 whereas for bulk is 2.3:1), which could allow for the detection of low levels of impurities that would otherwise be hidden in the background when considering the bulk mass spectrum. Peaks of Al^+^ and Al^2+^ are predominant both in the bulk and at the GB. Furthermore, the Al^3+^ peak, which is a sign that the electric field is locally slightly less elevated, thus often correlated with regions that are less affected by residual gas, is not observed in the GB mass spectrum. As a result, peaks pertaining to spurious species, such as O^+^, are not observed in significant amounts at the grain boundary. One possible explanation could be related to the low energy configuration of grain boundary. One possible explanation could be related to the low energy configuration of the grain boundary. The sensitivity that we can estimate from such a spectrum is in the range of 150 ppm, and yet, no specific trace elements located exclusively at the grain boundary appear, emphasizing the cleanliness of the sample and the low energy configuration of the Σ3(70.53°) [110](1-1-1) grain boundary [44].

## 5 Conclusions

In conclusion, BooT-PCA demonstrated its ability to extract information regarding the topography of grain boundaries from atom probe data by using the *Boosting* object tracker on features observed in the pattern formed on the detector. This new method for data extraction allows for filtering out both structural features, including a complete reconstruction of the grain boundary surface, as well as a composition distribution profile, with the possible extraction of atoms located specifically at the extracted feature within the atom probe data.

## 6 Appendix

### 6.1 TAPsim simulation

The simulation was executed on a two-socket Xeon Gold 6150 Ubuntu 16.04 workstation with 192 GB main memory. Using TAPSim in version 1.0b, rev3225, compiled with O3 optimization, and running six threads in parallel, enabled to achieve a net simulated evaporation rate of 142 atoms per minute.

The resulting virtual bi-crystal resembled closely our experimental specimens, to the exception of the relative position of the boundary within the virtual specimen and the specimen’s size. These are the results of a compromise. Specifically, we moved the boundary along the specimen’s main axis. Thereby it entered the tip closer to the initial apex to facilitate earlier detection on the detector compared a setup that would have simulated the entire specimen. Such setup of 48.4 × 10^6^ ions would have caused an impractical long and computational costly simulation. In fact, even for parallelized execution and assuming an optimistic evaporation rates (300 atoms per minute [34]) such simulation would have taken at least 112 days.
